# Transcutaneous Closure of Aortic Valve Cusp Perforation

**DOI:** 10.1016/j.shj.2022.100013

**Published:** 2022-04-04

**Authors:** Mike El Asmar, Dounia Z. Iskandarani, Walid Gharzeddine, Fadi J. Sawaya

**Affiliations:** Division of Cardiology, American University of Beirut Medical Center, Beirut, Lebanon

**Keywords:** Cusp perforation, Occluder device, Transcatheter closure

We report the case of a 58-year-old patient with history of mechanical mitral valve replacement 1 year ago due to *Staphylococcus aureus* endocarditis that resulted in severe mitral regurgitation. During follow-up, he had progressive heart failure symptoms, New York Heart Association class III, with severe hemolytic anemia. Transthoracic echocardiography revealed mildly dilated left ventricle and dilated left atrium with evidence of severe mitral paravalvular leak (PVL) and severe aortic regurgitation (AR). Transesophageal echocardiography (TEE) showed severe anterolateral mitral PVL (Figure 1) and severe AR due to perforation of the noncoronary cusp (NCC; Supplemental Video 1).

**Figure 1:**
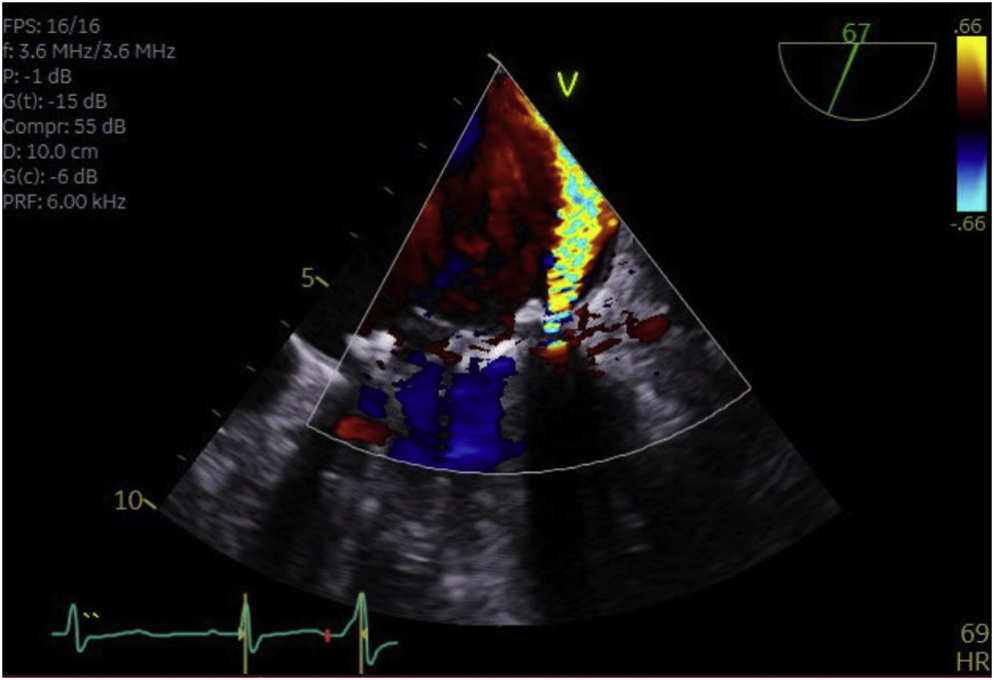
**Transesophageal echocardiography: Anterolateral mitral paravalvular leak**.

After refusing surgery, we decided to perform transcatheter mitral PVL device closure and to assess the AR perforation for possible intervention.

Under general anesthesia and TEE guidance, the mitral valve PVL was managed through a trans-septal approach. After crossing the leak with the aid of an Agilis Medium deflectable catheter, an 8F Cook shuttle sheath was advanced across the mitral PVL, and an AMPLATZER Vascular Plug (AVP) III 10/5 mm was deployed successfully with the resolution of the leak and significant decrease of the left atrial V wave, with no interference with the mechanical mitral leaflets (Supplemental Video 2).

A 5F sheath was then inserted in the right femoral artery. Aortogram showed significant AR with a significant jet coming from the NCC (Figure 2, Supplemental Video 3). The perforated site of the NCC was crossed with a Terumo wire (Figure 3, Supplemental Video 4). A multipurpose catheter was advanced through the perforated cusp (Supplemental Video 5), and an AVP IV 8 mm was deployed successfully (Supplemental Video 6), resulting in significant decrease in AR on TEE and fluoroscopy (Supplemental Videos 7 and 8). The AMPLATZER family we typically use for closure of leaks are the AVP II, AVP III, and AVP IV (Figure 4). The AVP II and III have a larger profile, are bulkier, and are considered a good choice for aortic/mitral PVL leaks depending on whether the defect is circular or crescentic. For the aortic perforation, we opted for the lowest profile and softest profile that goes through a 5F diagnostic catheter with 2 heads that seals on both sides. Given that the aortic valve will open and close all the time, we believed that the AVP IV will be the least traumatic device.^1^

**Figure 2:**
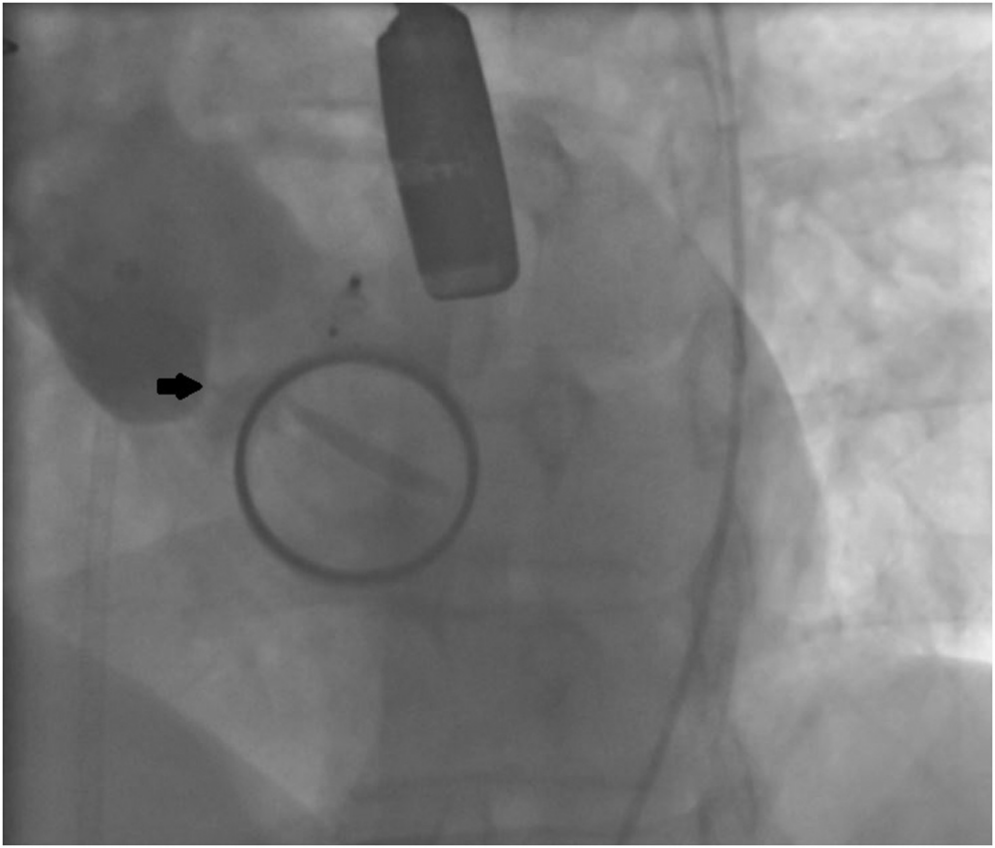
**Fluoroscopy: Aortic regurgitation from perforated noncoronary cusp**.

**Figure 3:**
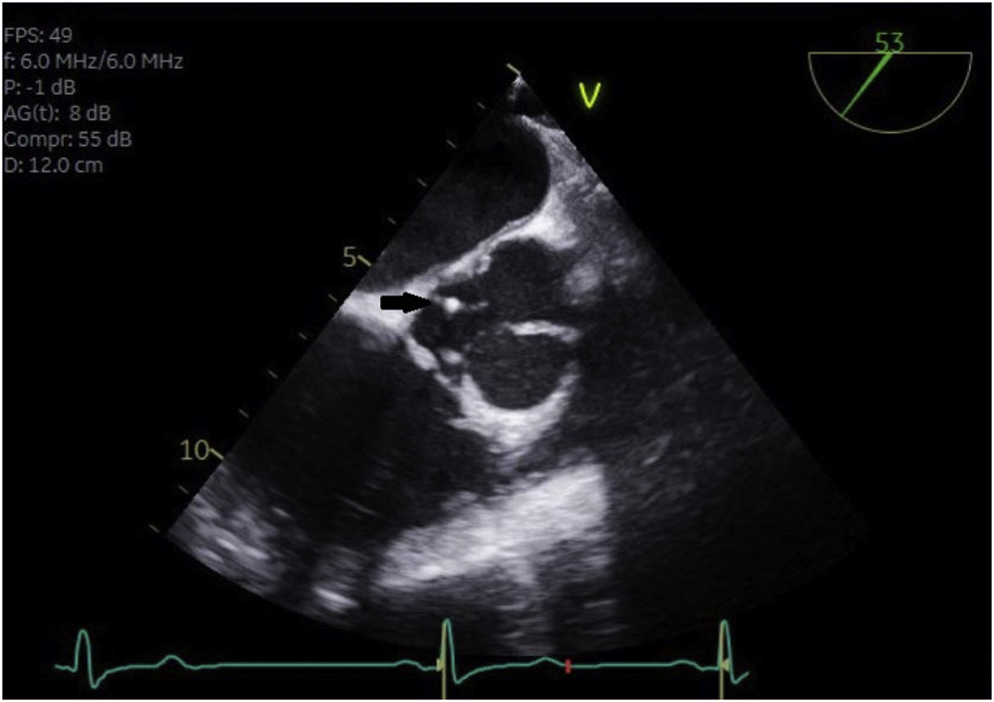
**Transesophageal echocardiography: Wire crossing the noncoronary cusp**.

**Figure 4:**
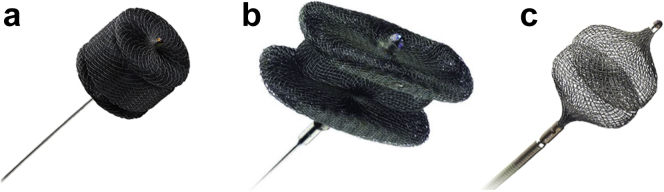
**Family of AMPLATZER Vascular Plugs.** (a) AVP II, (b) AVP III, and (c) AVP IV.

Aortic valve leaflet defects are usually managed by the surgical approach. To our knowledge, this is the first description of a retrograde closure of an aortic valve cusp perforation through percutaneous approach with the AVP IV device. One month after the procedure, our patient’s symptoms improved with resolution of the hemolytic anemia and back to New York Heart Association I class.

## Consent statement

Consent was obtained from the patient for publication of this report and any accompanying images.

## Funding

The authors have no funding to report.

## Disclosure statement

The authors report no conflict of interest.

## Supplementary Material

Supplemental Video 1 TEE: Systolic mitral PVL and diastolic AR from a perforated NCC.

Supplemental Video 2 TEE: AMPLATZER Vascular Plug III with significant reduction of mitral PVL.

Supplemental Video 3 Fluoroscopy: Aortic regurgitation from perforated NCC.

Supplemental Video 4 TEE: Wire crossing the perforated site of the NCC.

Supplemental Video 5 Fluoroscopy: Multipurpose catheter crossing the perforated site of NCC.

Supplemental Video 6 Fluoroscopy: Deployment of an AMPLATZER Vascular Plug IV in the NCC.

Supplemental Video 7 TEE: AMPLATZER Vascular Plug IV in place in the NCC with resolution of AR.

Supplemental Video 8 Fluoroscopy: Resolution of regurgitation post deployment of an AMPLATZER Vascular Plug IV in the NCC.
